# Audiological findings in Brown Vialetto-Van-Laere Syndrome: A scoping review

**DOI:** 10.1016/j.bjorl.2025.101558

**Published:** 2025-02-07

**Authors:** Débora de Oliveira Rolim, Leticia Cristina Vicente, Hugo Amilton Santos de Carvalho, Rebeca Lorrane Santana Santos, Rivadávio Fernandes Batista de Amorim, Fayez Bahmad

**Affiliations:** a Universidade de Brasília (UnB), Brasília, DF, Brazil; b Instituto Brasiliense de Otorrinolaringologia, Brasília, DF, Brazil

**Keywords:** Progressive bulbar palsy with sensorineural deafness, BrownVialetto-Van Laere Syndrome, Audiometries, Audition

## Abstract

**Objective:**

This study aimed to characterize audiological porfile in inviduals with Brown-Vialetto-Van Laere syndrome (BVVLS).

**Methods:**

This is a scoping review following the methodological structure developed by the Joana Briggs Institute (JBI). The PCC mnemonic was used to elaborate the research question, which resulted in the research question: “What are the audiological findings in individuals with BVVLS?”.

**Results:**

All of the studies included in this review were case reports. The main audiological findings are sensorineural hearing loss and Auditory Neuropathy Spectrum Disorder (ANSD).

**Conclusion:**

All individuals presented a severe to profound bilateral hearing loss, related to ANSD.

## Highlights

• BVVL Syndrome is a rare alteration in the Riboflavin transporter gene.
• Few studies describe the details of hearing loss in the syndrome.
• The main finding was sensorineural hearing loss.
• Objective tests were integrated into the audiological diagnosis process.

## Introduction

Brown-Vialetto-Van Laere Syndrome (BVVLS) is a rare alteration in the Riboflavin transporter gene which is a water-soluble vitamin from the B complex (B2). Mutations in the SLC52A3 gene (also known as BVVLS, BVVLS1, C20orf54, RFT2, RFVT3, bA371L19.1, hRFT2) are associated with the neurological and motor disorders characteristic of BVVLS, occurring mainly with progressive ponto-bulbar palsy and hearing loss.^1^

The disease presents with VII, IX, X, XI, and XII cranial nerve palsies, which develop over a relatively short period of time in a previously healthy individual. The disease is characterized by its progressive nature, including hearing implications, due to alterations in the metabolism of amino acids, fatty acids and purines.^2^, ^3^

According to the available literature, the article with the largest number of individuals studied in the last 100 years reported that in the vast majority of cases the first symptom was sensorineural deafness, which is usually progressive and severe.^4^ The time between the onset of deafness and the development of other symptoms has been reported to be shorter in males (mean of approximately 5 years) than in females (mean of almost 11 years).^4^, ^5^ Very rarely, affected cases do not appear to develop deafness, presumably because these individuals die before the hearing impairment develops.^5^

Sensorineural hearing loss may precede the neurological signs,^6^ for this reason, audiological monitoring in these cases becomes highly relevant and is essential for early intervention. Such management can considerably minimize the impact of these changes on the quality of life of these individuals.^7^ Although some studies correlate hearing loss with the syndrome, few describe the hearing assessment process and its outcome. Therefore, this study aimed to characterize audiological profile in individuals with BVVLS.

## Methods

This is a scoping review of the literature, following the methodological structure developed by the Joana Briggs Institute (JBI).^8^ The study consists of five phases: identification of the research question, identification of relevant studies, study selection, data mapping, grouping, summarizing, and reporting results.^9^

### Preparation of the research question

The PCC mnemonic was used to elaborate the research question (P: Population, C: Concept, C: Context), with “P” being the population (individuals with Brown Vialetto Van Laere syndrome), “C” being the concept (audiological profile), and “C” the context (diagnostic hearing assessment) which resulted in the research question: “What is the audiological profile in individuals with BVVLS?”. The descriptors to compose the search strategies were selected from Medical Subject Headings (MeSH) and combined using the Boolean operators “OR” and “AND”.

### Eligibility criteria

As it is a rare syndrome, all studies published in scientific journals that described hearing thresholds by frequency individually or as an average were considered, as long as the classification used was included, in individuals with BVVLS. There was no limitation regarding the year of publication and language.

Studies that had as main focus describing purely genetic characteristics of the syndrome, reviews, opinion articles, and conference abstracts were excluded.

### Information sources

On November 10, 2023, the detailed search strategy was applied to the PubMed, Scopus, and Web of Science databases, as well as Google Scholar, to search for gray literature (Table 1). To extract relevant data, the following variables were used: title, authors, language, periodical, year of publication, type of publication, objective and results.

**Table 1:** Search strategy applied in databases.

Data base	Search Strategy
PubMed	("Progressive Bulbar Palsy with Sensorineural Deafness" [All Fields] OR "Pontobulbar Palsy With Deafness" [All Fields] OR "Pontobulbar palsy and neurosensory deafness" [All Fields]) AND ("Audiometries" [All Fields] OR "Speech Audiometries" [All Fields] OR "Speech Audiometry" [All Fields] OR "Pure Tone Audiometry" [All Fields] OR "Pure-Tone Audiometry" [All Fields] OR "Bekesy Audiometry" [All Fields] OR "Evoked Response Audiometry"[All Fields] OR "Electrocochleography" [All Fields] OR "Acoustic Impedance Test" [All Fields] OR "Impedance Audiometry" [All Fields] OR "Tympanometry" [All Fields] OR "Spontaneous Otoacoustic Emission" [All Fields] AND "Hearing"[All Fields] OR "Audition" [All Fields])
Scopus	(ALL ("Progressive Bulbar Palsy with Sensorineural Deafness" OR "Pontobulbar Palsy With Deafness" OR "Pontobulbar palsy and neurosensory deafness" OR "BrownVialetto-Van Laere syndrome") AND ALL ("Audiometries" OR "Speech Audiometries" OR "Speech Audiometry" OR "Pure Tone Audiometry" OR "Pure-Tone Audiometry" OR "Bekesy Audiometry" OR "Evoked Response Audiometries" OR "Evoked Response Audiometry" OR "Audiometries Electroencephalic Response" OR "Electroencephalic Response Audiometry" OR "Electrocochleography" OR "Electrocochleographies" OR "Acoustic Impedance Test" OR "Impedance Audiometry" OR "Tympanometry" OR "Electroacoustic Impedance Tests" OR "Spontaneous Otoacoustic Emission") AND ALL ("Hearing" OR "Audition"))
Web of Science	“Progressive Bulbar Palsy with Sensorineural Deafness” OR “Pontobulbar Palsy With Deafness” OR “Pontobulbar palsy and neurosensory deafness” OR "BrownVialetto-Van Laere syndrome" (All Fields) and “Hearing” OR “Audition” (All Fields)
Google Scholar	“Progressive Bulbar Palsy with Sensorineural Deafness” AND “Audiometries” OR “Speech Audiometries” OR “Speech Audiometry” OR “Pure Tone Audiometry” OR “Pure-Tone Audiometry” OR “Bekesy Audiometry” OR “Evoked Response Audiometries” OR “Evoked Response Audiometry” OR “Audiometries Electroencephalic Response” OR “Electroencephalic Response Audiometry” OR “Electrocochleography” OR “Electrocochleographies” OR “Acoustic Impedance Test” OR “Impedance Audiometry” OR “Tympanometry” OR “Electroacoustic Impedance Tests” OR “Spontaneous Otoacoustic Emission”

### Data organization and analysis

A blind analysis with two judges of the methodological quality of the case report articles included in this review was carried out using the CARE-Checklist instrument.^10^ The Kappa level of concordance was classified as substantial for all the studies included.^11^ The judges agreed that all the included studies were ethical and complied with all the requirements regarding the methodology of a case report.

Based on the results obtained, tables were created and discussed qualitatively and descriptively.

## Results

**Fig. 1:**
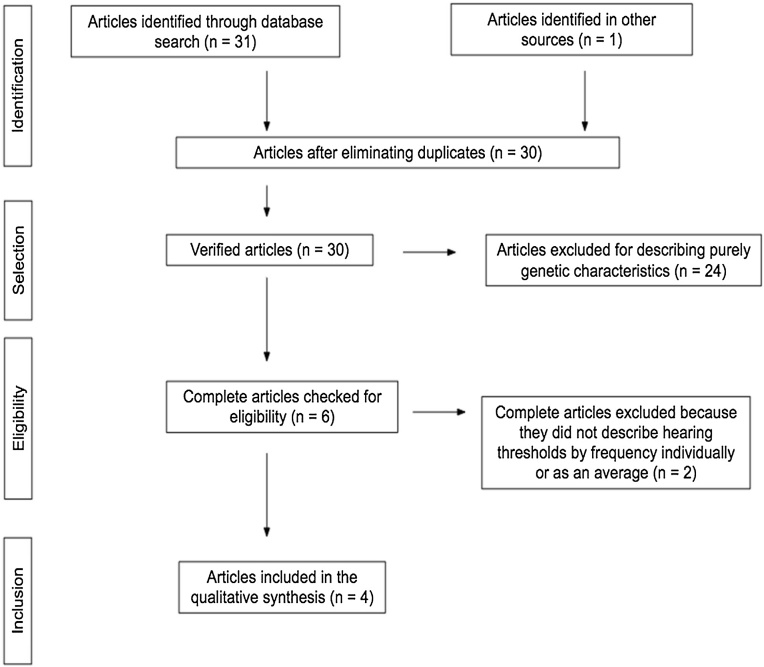
Flowchart adapted from PRISMA.

The search and selection process for studies in this review is presented in the flowchart (Fig. 1) according to JBI recommendations.^12^

All of the studies included in this review were case reports. Among the main audiological findings are sensorineural hearing loss, Auditory Neuropathy Spectrum Disorder (ANSD), and auditory rehabilitation using Cochlear Implants (CI), which can be observed in Table 2.

**Table 2:** Included studies and their main audiological findings.

Author and Year	Type of study	Casuistry	Audiological Exams Performed	Audiological Findings
Piecuch, Skarżyński and Skarżyński (2023^13^	Case report	Female, 4 years old	Audiometry in free field with behavioral observation	Bilateral profound sensorineural hearing loss
			Brainstem auditory evoked potential	Auditory neuropathy spectrum disorder
			Tympanometry	Hearing rehabilitation with Cochlear Implant
			Otoacoustic Emissions	
Alasqah et al. (2023^14^	Case report	Male, 1 year old	Audiometry in free field and with visual reinforcement	Bilateral profound sensorineural hearing loss
			Brainstem auditory evoked potential	Auditory neuropathy spectrum disorder
			Otoacoustic emissions	Hearing rehabilitation with cochlear implant
do Amaral et al. (2022^15^	Case report	Female, 31 years old	Pure Tone Audiometry	Moderately severe bilateral sensorineural hearing loss
			Speech Perception Test	
			Brainstem auditory evoked potential	Auditory neuropathy spectrum disorder
			Otoacoustic emissions	Hearing rehabilitation with cochlear implant
Mutlu, Topcu and Ciprut (2019^16^	Case report	Male, 6 years old	Conditioned audiometry in free field	Bilateral profound sensorineural hearing loss
			Brainstem auditory evoked potential	Auditory Neuropathy Spectrum Disorder
			Otoacoustic emissions	Riboflavin Therapy for Treatment of Hearing Loss
			Tympanometry	
			Steady-state auditory evoked potential	

In all the case reports included in this review, pathologies such as hearing loss,^13^, ^14^, ^15^, ^16^ breathing difficulties^14^ and reduced laryngeal mobility^13^ were described as the first symptoms of BVVLS.

## Discussion

Among the audiological findings, profound sensorineural hearing loss was the most common characteristic among such individuals and is directly correlated with mutations in the SLC52A2 or SLC52A3 genes, already described in the literature.^17^ Auditory Neuropathy Spectrum Disorder (ANSD) was also an important finding, which, in addition to severe or profound sensorineural hearing loss, can involve aspects that significantly impact the acquisition and development of auditory skills, such as the discrimination of sounds from speech.^18^

SLC52A2 and SLC52A3 mutations reduce riboflavin transporter protein causing an imbalance in the cellular metabolism of tissues that need to absorb riboflavin (vitamin B2 complex), because the body does not produce vitamin B2 endogenously, it is acquired through the diet. The RFVT2 transporter is expressed mainly in the brain and spinal cord, while RFVT3 is expressed in high levels in the small intestine. Alteration in the functionality of these riboflavin transporters is directly related to the neuronal dysfunction observed in individuals with BVVLS,^19^ this may be strongly related to the audiological finding of (ANSD) observed in all the case reports included in this review.

Audiological diagnostic criteria for ANSD are well accepted. Traditional clinical tests classically reveal present otoacoustic emissions, absent or abnormal auditory brainstem response with the record of cochlear microphonic, absent or elevated middle ear muscle reflex and impairment of speech perception that is incompatible with hearing threshold,^20^, ^21^ which can be variable in these individuals.^20^

In the studies included, audiological assessments through subjective tests such as tonal audiometry were performed with different strategies adapted to the inherent characteristics of each individual, corroborating essential studies in the field of audiological assessment.^22^ In all studies analyzed, objective tests such as Brainstem Auditory Evoked Potential and Steady-State Auditory Evoked Potential, Otoacoustic Emissions, tympanometry and acoustic reflexes measurement were also integrated into the audiological diagnosis process of these individuals; when correlated with each other can ensure a more assertive assessment, as recommended by the crosscheck principle of audiological assessment.^23^ Although the subjects' ages ranged in years from 1 to 31, it was possible to assess each one using techniques and tests appropriate to their age group. It is therefore essential to carry out audiological assessment on these individuals.

In one of the studies, the treatment of hearing loss was carried out through Riboflavin replacement, a treatment also reported by Menezes et al. (2016^24^ who analyzed the results of audiological tests pre and post-Riboflavin therapy of 7 individuals with BVVLS. For 2 individuals, it was observed improvement in auditory thresholds in the pure tone audiometry and for one individual, the mild hearing loss in low frequencies reached normal values after 6 months of treatment. However, the improvement in tonal hearing thresholds was not enough to promote good discrimination of speech sounds after 12 months of treatment, so, it was decided to rehabilitate hearing through CI surgery.^25^

This finding corroborates the study by do Amaral et al. (2022), suggesting that the difficulty in auditory discrimination for speech sounds in individuals with ANSD is independent of tonal auditory thresholds. Therefore, when poor results are maintained despite adequate trial with appropriately fitted amplification, cochlear implantation should be considered, regardless of audiometric thresholds.^21^

Studies reported that after CI surgery, individuals with auditory neuropathy spectrum disorder improved their performance of hearing skills and had similar performance to that of individuals with sensorineural hearing loss using CI.^26^ In our review, improvement in auditory thresholds was reported in two individuals with BVVLS, the first case^13^ reported that post-implantation effects were assessed 4 and 5 months after implantation at the level of progression from profound to moderate hearing loss in the right ear and severe hearing loss in the left ear. In another case,^15^ it was reported that after 5 years of the first surgery, the patient presented a mean of 28 dB of tone threshold in an open field, using bilateral CI devices. In speech perception, there was a 64% of auditory detection of dissyllables and 100% of trisyllables, both in open set. Considering this is a viable rehabilitation option for these patients, it is necessary to report on their performance using the CI in order to understand the particularities of monitoring and counseling patients and their families on the real expectations about the benefits of CI.

Even though hearing loss is common in this population, only a few studies described hearing results in a more detailed way, hence this aspect is considered a significant limitation in the selection process of studies to be included in this review. Therefore, the audiological profile may be more variable that the one related in this review, more studies are needed to report these results.

## Conclusion

Audiological assessment in individuals with BVVLS is possible in a vast age range using appropriate techniques. Results revealed a severe to profound bilateral hearing loss in all individuals, related to ANSD. Rehabilitation through CI occurred in most cases, although there is still no information about the language outcomes in these individuals for the time being.

Financial disclosures

This research did not receive any specific grant from funding agencies in the public, commercial, or not-for-profit sectors.

## Declaration of competing interest

The authors declare no conflicts of interest.
